# Supplementary material for: Giant piezoresistive effect by optoelectronic coupling in a heterojunction
Source: Nat Commun. 2019 Sep 12;10:4139. doi: 10.1038/s41467-019-11965-5 (PMC6742666; doi:10.1038/s41467-019-11965-5)
Supplement: Supplementary file 1 — Supplementary Information [file 41467_2019_11965_MOESM1_ESM.pdf]

Supplementary Information for

Giant piezoresistive effect by optoelectronic coupling in a heterojunction

Thanh Nguyen[1], Toan Dinh[1], Abu Riduan Md Foisal[1], Hoang-Phuong Phan[1], Tuan-Khoa Nguyen[1], Nam-Trung Nguyen[1], and Dzung Viet Dao[1,2]

[1]Queensland Micro- and Nanotechnology Centre, Griffith University, Queensland, Australia. [2]School of Engineering and Built Environment, Griffith University, Queensland, Australia. Correspondence and requests for materials should be addressed to D.V.D. (email: d.dao@griffith.edu.au) or to T.D. (email: toan.dinh@griffith.edu.au) or to T.N. (email: thanh.nguyen11@griffithuni.edu.au)

Supplementary Figures

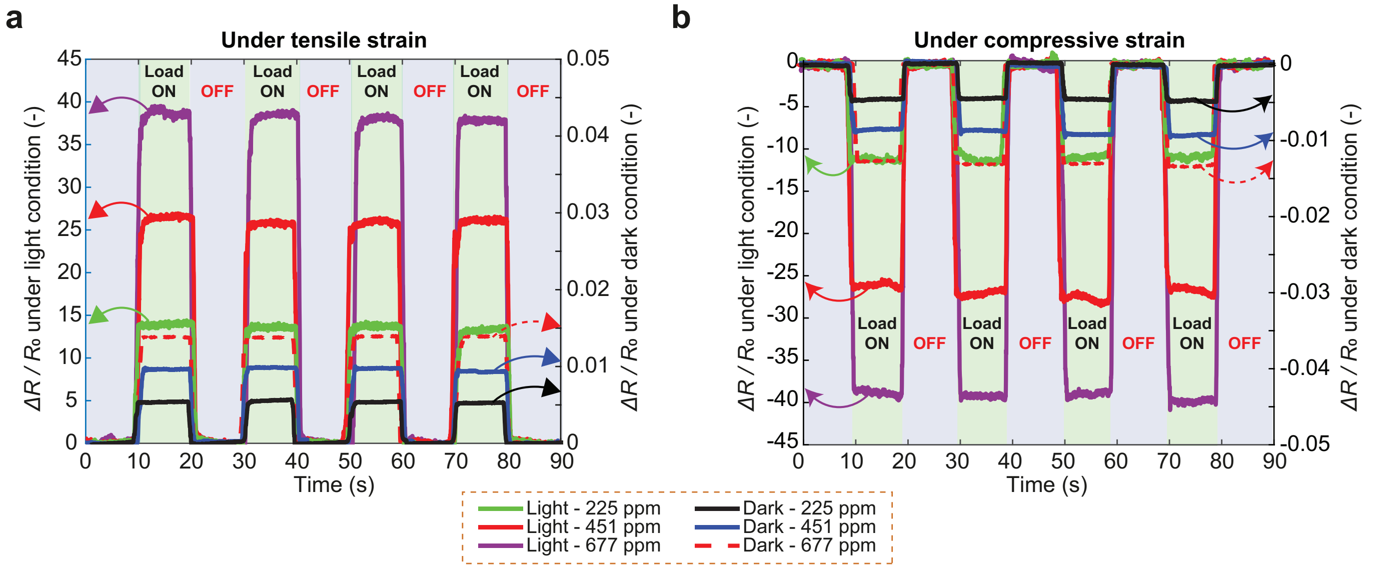

Supplementary Figure 1: Repeatability of the fractional change in resistance under light and dark conditions. (a) Repeatability of the fractional change as different tensile strains were periodically applied (i.e., Load ON) and released (i.e., Load OFF) in the cantilevers. **(b)** Repeatability of the fractional change as different compressive strains were periodically applied (i.e., Load ON) and released (i.e., Load OFF) in the cantilevers.

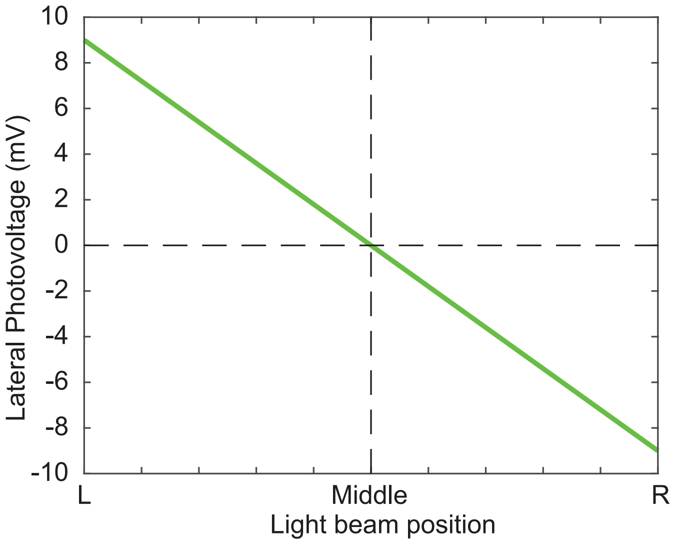

Supplementary Figure 2: Dependence of the photovoltage on the light position. Light beam with intensity of 19,000 lux was positioned from electrode L to electrode R, the output voltage was measured. As the light beam was at electrode L, the lateral photovoltage was approximately 9 mV. This photovoltage was -9 mV when the light beam was at electrode R, and the value was 0 V when the light beam shining at the middle between the two electrodes (L and R).

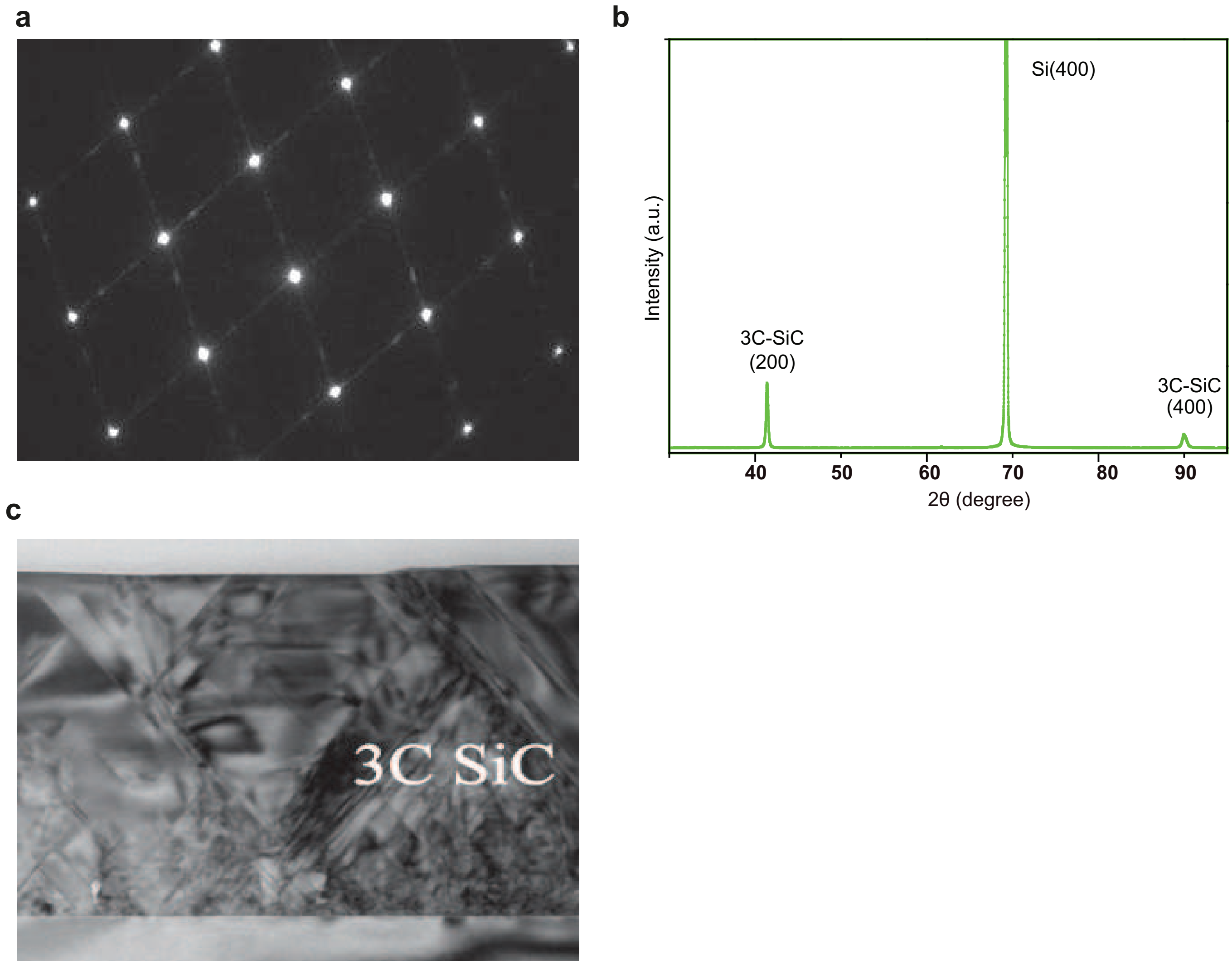

Supplementary Figure 3: The characteristics of single crystalline 3C-SiC grown on a single crystalline Si substrate. (a) The selected area electron diffraction image of the single crystalline 3C-SiC. **(b)** X-ray diffraction graph of the single crystalline 3C-SiC grown on the single crystalline Si substrate. **(c)** The transmission electron microscopy image of the single crystalline 3C-SiC on the Si substrate.[1]

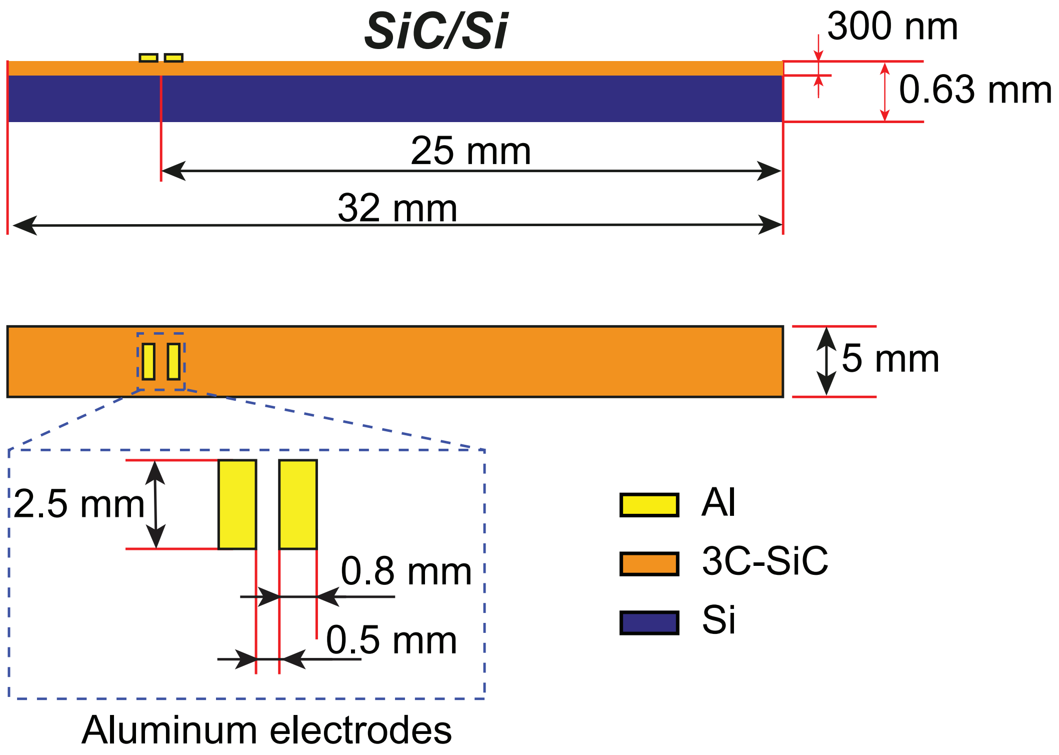

Supplementary Figure 4: Geometry of the cantilevers. The distance from the piezoresistor to the free end of the cantilever was 25 mm, and the whole length of the cantilever was 32 mm. The width and thickness of the cantilever were 5 mm and 0.63 mm, respectively. The thickness of the SiC layer was 300 nm. The dimensions of the piezoresistor were 0.5 mm $\times$ 2.5 mm.

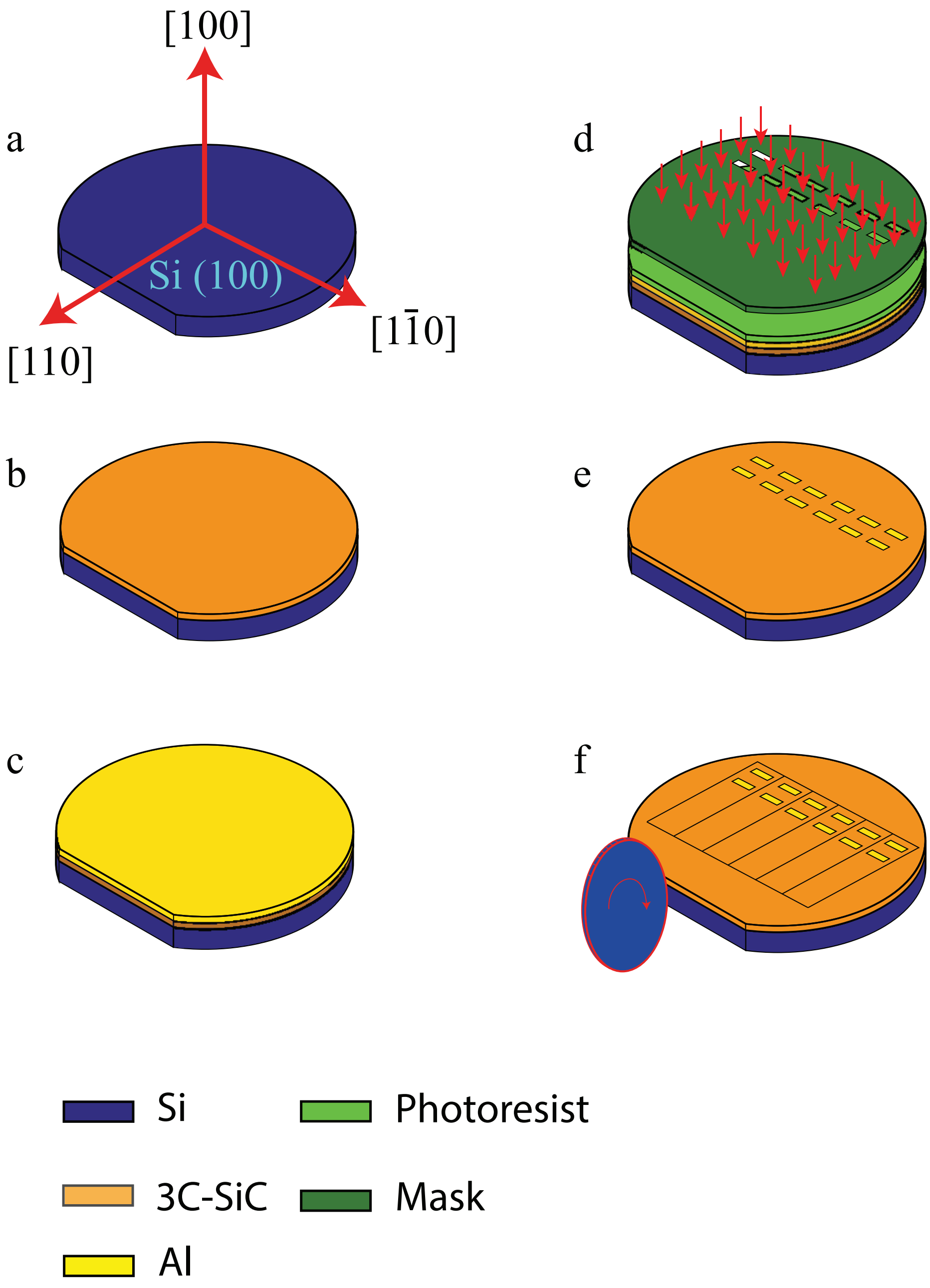

Supplementary Figure 5: Fabrication process of the 3C-SiC/Si cantilever. (a) Si wafer (100) preparation, **(b)** growth of 3C-SiC on a Si wafer by low pressure chemical vapour deposition (LPCVD) method at a temperature of 1,000°C, **(c)** deposition of aluminium on the surface of the 3C-SiC nanofilm, **(d)** photolithography, **(e)** wet etching aluminium, **(f)** dice of the 3C-SiC/Si wafer. The fabrication process of the 3C-SiC/Si cantilever started from a p-type Si wafer (100), which have a doping concentration of $5 \times 10^{14}\ cm^{-3}$. A 3C-SiC nanofilm with the thickness of 300 nm was epitaxially grown on the (100) Si substrate. After that, two aluminium electrodes were deposited and patterned on the 3C-SiC surface. Finally, the 3C-SiC/Si wafer was diced to create the cantilevers (32 mm $\times$ 5 mm $\times$ 0.63 mm). Ohmic contacts between the electrodes and the 3C-SiC layer have been demonstrated.

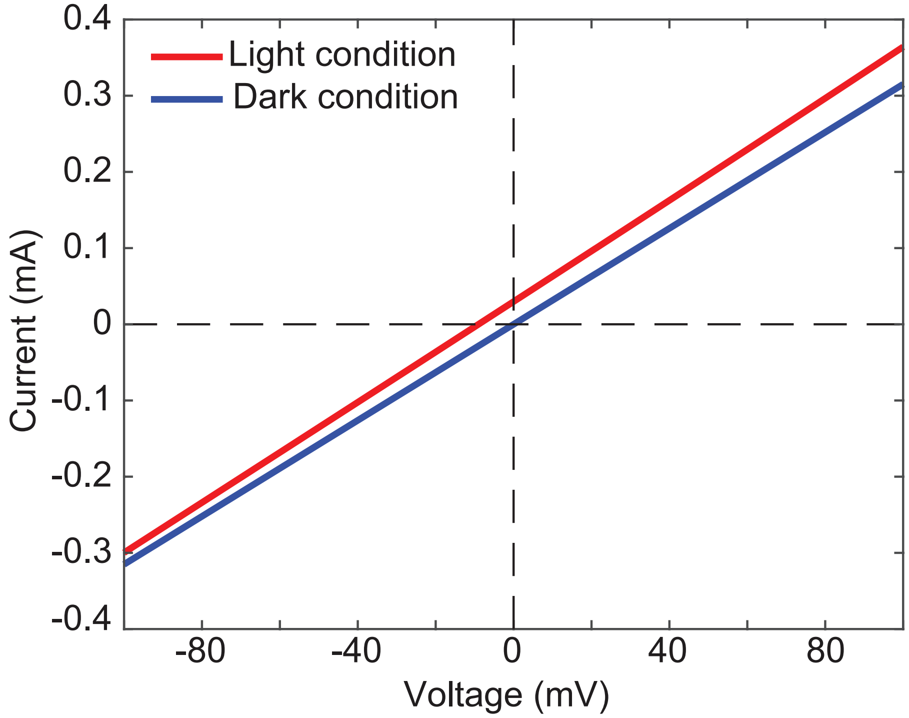

Supplementary Figure 6: The current-voltage characteristics under dark and light conditions in strain-free condition. The I-V curves were linear under both light and dark conditions.

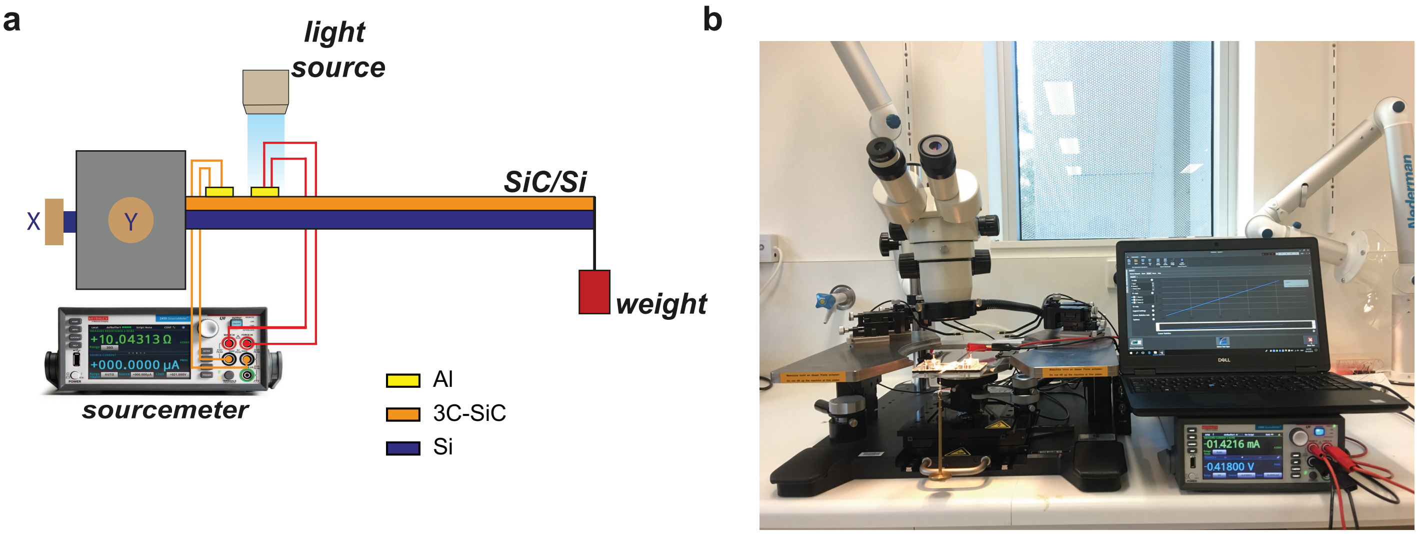

Supplementary Figure 7: Experimental setup. (a) The brief schematic of the experiment. **(b)** A photography of the experiment. In our experiment, the cantilevers were mounted on the chuck of the EP 6 CascadeMicrotech probe system. The cantilevers were illuminated by light from the Fibre Optic Illuminator of the EP 6 CascadeMicrotech probe system. The position of the light beam could be adjusted in three directions, and the light intensity could be regulated by changing supply power. A Keithley 2450 SourceMeter was used to control the electrical current and simultaneously measure the output voltage. Three weights of 50 g, 100 g, 150 g were used to induce strains in the cantilever.

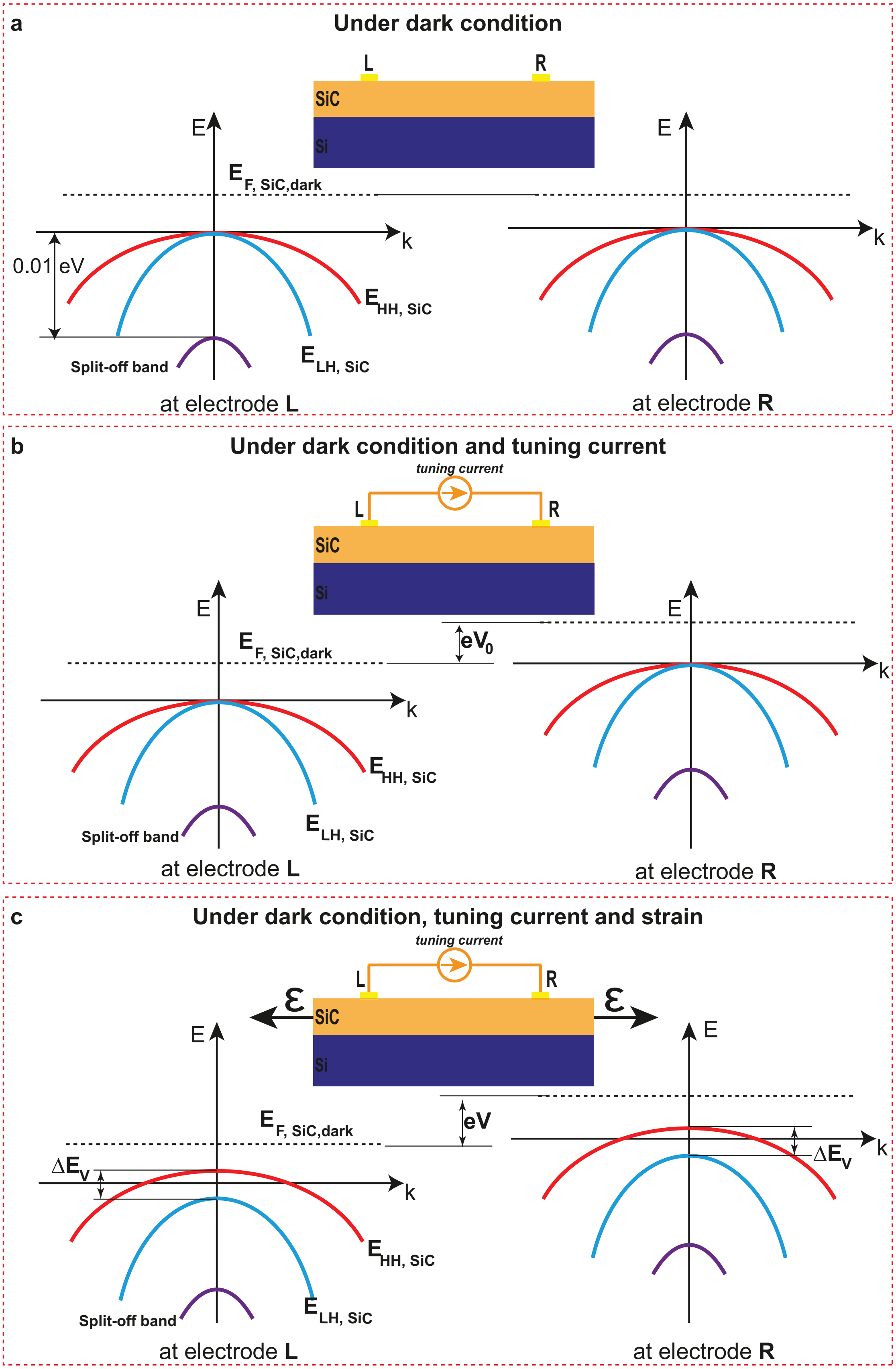

Supplementary Figure 8: E-k (energy-momentum) characteristics of 3C-SiC nanofilms under dark condition. (a) E-k characteristics at electrode L and electrode R under dark condition, **(b)** under dark condition and tuning current, **(c)** under dark condition, tuning current and applied strain. Under dark condition, the E-k diagrams of SiC at the two electrodes are the same. When an electrical current is supplied to the two electrodes, the band energy is bend upwards from electrode L to electrode R, which results in a difference of the Fermi levels $\Delta E = eV_0$ between the two electrodes, where V_0 is the output voltage without strain, which is much larger than that under light conditions.

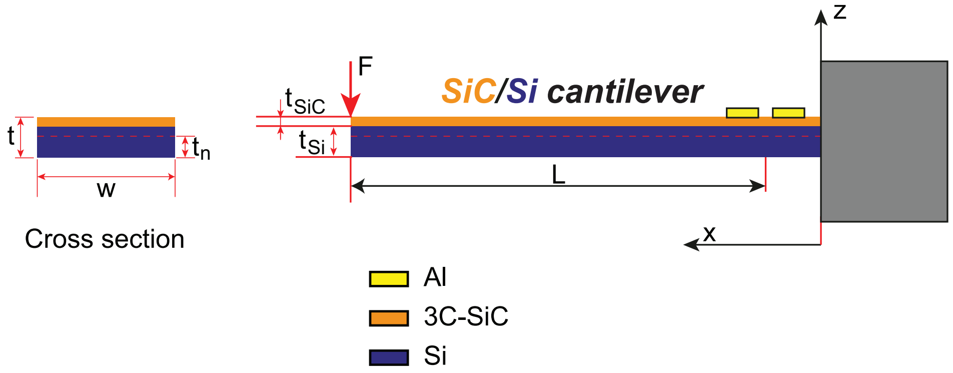

Supplementary Figure 9: A cantilever with load at the free end.

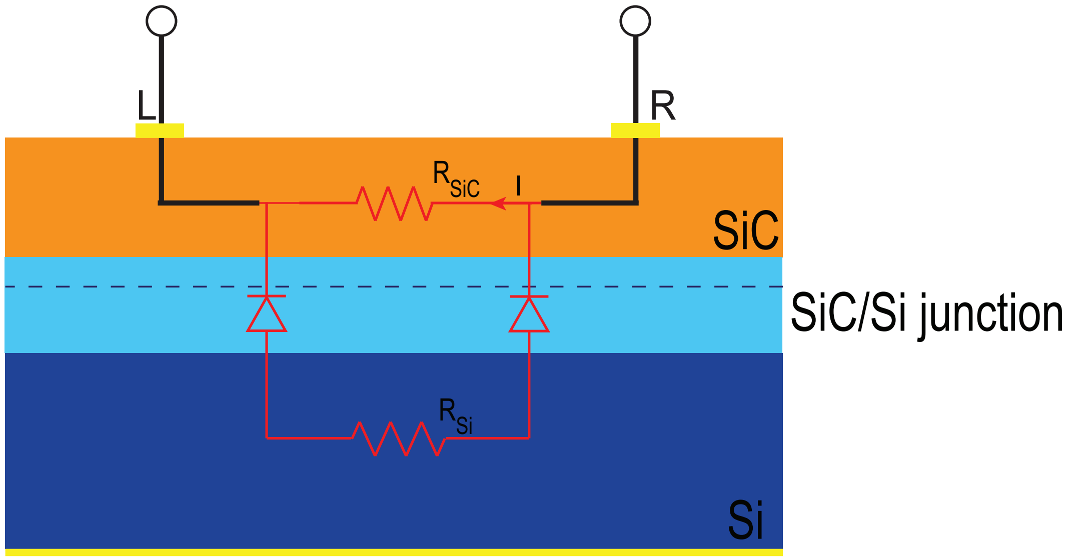

Supplementary Figure 10: Equivalent circuit model of the sample.

Supplementary Tables

Supplementary Table 1: Strain calculation results

Load (g)	F (mN)	w (mm)	t (mm)	L (mm)	E_{Si} (GPa)	E_{SiC} (GPa)	ε (ppm)
50	491	5	0.63	25	170	330	225
100	982	5	0.63	25	170	330	451
150	1473	5	0.63	25	170	330	677

Supplementary Notes

Supplementary Note 1: Strain calculation

Supplementary Figure 9 depicts a cantilever with one end clamped and one end free. The width and thickness of the cantilever are w and t, respectively. The distance from the free end (load point) to the centre of the piezoresistor is L. t_{Si} and t_{SiC} are the thickness of the Si substrate and SiC thin film, respectively. E_{Si} and E_{SiC} are Young's moduli of Si and SiC in the [100] orientation. A force F is applied to the free end of the cantilever.

The strain ε in the centre of the piezoresistor was calculated using a bending model of a bi-layer beam as SiC was epitaxially grown on the Si substrate with assumption that the bonding between the Si substrate and SiC layer is perfect. As the lengths of the Si substrate and SiC layer are equal, the lateral strain of the piezoresistor is [2]

$$\varepsilon = \frac{F}{wD} L t_n = \frac{F}{wD} L \frac{t}{2} \tag{1}$$

where t_n is the distance from the neutral axis to the piezoresistor. The bending modulus per unit is estimated as

$$D = \frac{E_{Si}^2 t_{Si}^4 + E_{SiC}^2 t_{SiC}^4 + 2E_{Si}E_{SiC}t_{Si}t_{SiC}(2t_{Si}^2 + 3t_{Si}t_{SiC} + 2t_{SiC}^2)}{12(E_{Si}t_{Si} + E_{SiC}t_{SiC})} \tag{2}$$

Substitute the given parameters into equations 1 and 2, we can find strain at the sensing element corresponding with three applied loads of 50 g, 100 g, and 150 g as shown in Supplementary Table 1. We have also confirmed these results using finite element analysis (FEA) method.

Supplementary Note 2: Equivalent circuit model

In this research, the p-Si substrate and heterojunction play critical roles in the generation and redistribution of charge carriers (electron/hole pairs) into the 3C-SiC thin film. The sensing element was the SiC thin film resistor defined by two electrodes. The equivalent circuit model is shown in Supplementary Figure 10. The diode configuration of the heavily doped p-type 3C-SiC/p-type Si heterojunction only allowed the charge carriers to move from the Si side to SiC whenever there were excessive charge carriers in Si (e.g., by photon excitation). Therefore, this heterojunction configuration works well either when the Si substrate is floated or kept at a potential lower than the potential on the SiC side to maintain the reverse-biased condition. This concept was demonstrated by experiments in both cases (i.e., The Si substrate was grounded and floated.), and the results were similar.

Supplementary References

1. Md Foisal AR, et al. Pushing the limits of piezoresistive effect by optomechanical coupling in 3C-SiC/Si heterostructure. *ACS applied materials & interfaces* **9**, 39921-39925 (2017).
2. Gao X, Shih W-H, Shih WY. Induced voltage of piezoelectric unimorph cantilevers of different nonpiezoelectric/piezoelectric length ratios. *Smart Materials and Structures* **18**, 125018 (2009).
